# Effect of pulsed field ablation delivered from noncontact catheter electrodes on hemolysis: A tissue proximity indication–based analysis

**DOI:** 10.1016/j.hroo.2025.09.002

**Published:** 2025-09-08

**Authors:** Shingo Yoshimura, Kenichi Kaseno, Akiko Kodama, Suguru Nishiuchi, Kojiro Hattori, Taiki Masuyama, Takehito Sasaki, Kohki Nakamura, Shigeto Naito

**Affiliations:** Division of Cardiology, Gunma Prefectural Cardiovascular Center, Maebashi, Gunma, Japan

**Keywords:** Atrial fibrillation, Pulsed field ablation, Hemolysis, Variable-loop circular catheter, Tissue proximity indication

## Abstract

**Background:**

Pulsed field ablation (PFA) for atrial fibrillation can induce hemolysis, particularly when pulsed field (PF) energy is delivered from catheter electrodes that are not in contact with myocardial tissue. The variable-loop circular catheter (VLCC) incorporates tissue proximity indication (TPI) software to identify catheter-tissue contact. Whether PFA applications delivered from TPI-negative (noncontact) VLCC electrodes contribute to hemolysis remains unclear.

**Objective:**

The purpose of this study was to determine whether PFA delivered from TPI-negative VLCC electrodes is associated with postprocedural hemolysis.

**Methods:**

We retrospectively analyzed 20 consecutive patients who underwent their first PFA using the VLCC and CARTO 3 system (Biosense Webster Inc.). For each 3-pulse application set, catheter-tissue contact was assessed using TPI. We counted the cumulative number of VLCC electrode uses delivering PF energy; among these, those that were TPI-negative were summed and defined as the cumulative number of TPI-negative electrode uses (nTPI-EU). Postoperative lactate dehydrogenase (LDH), total bilirubin (T-Bil), and the postoperative-to-preoperative haptoglobin ratio served as hemolysis markers.

**Results:**

A mean of 78.3 ± 20.0 PFA applications (772.9 ± 204.1 VLCC electrode uses) were delivered per patient; the mean nTPI-EU was 438.8 ± 238.1. Compared with preoperative values, postoperative LDH and T-Bil increased significantly whereas haptoglobin decreased (*P* < .01 for all). nTPI-EU correlated positively with LDH (*r* = 0.663; *P* < .01) and T-Bil (*r* = 0.736; *P* < .01) and negatively with the postoperative-to-preoperative haptoglobin ratio (*r* = −0.556; *P* = .01).

**Conclusion:**

The cumulative number of noncontact catheter electrodes delivering PF energy was associated with hemolysis. Strategies that minimize the number of noncontact catheter electrodes, in addition to limiting total PFA applications, may reduce the risk of hemolysis.

**Key Findings**

▪ In pulsed field ablation (PFA) for atrial fibrillation, both inadequate catheter-tissue contact and a high number of PFA applications contribute to hemolysis.
▪ The cumulative number of noncontact electrode uses delivering pulsed field energy, as identified by the tissue proximity indication, correlated significantly with hemolysis markers.
▪ Minimizing the number of noncontact electrodes, in addition to limiting total PFA applications, may reduce the risk of hemolysis.

## Introduction

In recent years, pulsed field ablation (PFA) has been increasingly adopted for atrial fibrillation (AF).^1^ PFA induces irreversible electroporation: high-voltage electric fields delivered over an extremely short duration create pores in the cell membrane, ultimately leading to cell death.^2^ A major advantage of PFA is its tissue selectivity. Cardiomyocytes have significantly lower electroporation thresholds than surrounding nerves, vascular smooth muscle cells, and endothelial cells. This allows selective targeting of myocardial tissue while minimizing injury to critical adjacent structures, such as the esophagus, phrenic nerve, and pulmonary vein (PV) walls.^3^, ^4^, ^5^

However, PFA can induce hemolysis through the destruction of erythrocytes and, in rare cases, may result in acute kidney injury (AKI).^3^^,^^6^, ^7^, ^8^, ^9^ The degree of hemolysis increases with the number of PFA applications.^10^, ^11^, ^12^ Furthermore, animal experiments have shown that hemolysis is exacerbated when pulsed field (PF) energy is delivered from catheter electrodes that are not in contact with the myocardium.^13^, ^14^, ^15^

In clinical practice, both inadequate catheter-tissue contact and a high number of applications are thought to contribute to hemolysis. Achieving complete contact between all catheter electrodes and the myocardium remains technically challenging. The TRUPULSE generator (Biosense Webster Inc.) delivers short-duration, high-voltage, bipolar biphasic pulses to a variable-loop circular catheter (VLCC; VARIPULSE, Biosense Webster Inc.). This system incorporates tissue proximity indication (TPI) software, which monitors catheter-tissue contact in real time. A ≥7% increase in impedance from a baseline value measured while the electrode is in the blood pool is defined as TPI-positive and is displayed on the CARTO 3 three-dimensional electroanatomic mapping system (Biosense Webster Inc.).^16^ Saito et al^17^ reported that the mean percentage of TPI-positive electrode sites was 62% ± 27%, suggesting that a nonnegligible portion of PF energy may be delivered from catheter electrodes not in contact with myocardial tissue. Whether such PFA applications in the blood pool contribute to hemolysis has not yet been fully investigated. Therefore, the present study aimed to evaluate the impact of PFA applications delivered from noncontact catheter electrodes, identified using the TPI function, on postprocedural hemolysis.

## Methods

### Study population

This retrospective study included consecutive patients who underwent their first AF ablation using the VLCC and CARTO 3 system at Gunma Prefectural Cardiovascular Center between December 2024 and April 2025. Patients were excluded if periprocedural blood test data were unavailable. The study protocol was approved by the Ethics Committee of Gunma Prefectural Cardiovascular Center, and informed consent was obtained via an opt-out process. The study was conducted in accordance with the principles of the Declaration of Helsinki.

### Ablation protocol

Before the procedure, all patients underwent contrast-enhanced computed tomography or magnetic resonance imaging to assess left atrial (LA) anatomy. Deep sedation was achieved with propofol and dexmedetomidine; no neuromuscular blockers were administered. The procedures were performed by 3 operators. A multipolar catheter was introduced into the coronary sinus via the femoral vein, followed by a transseptal puncture. Intravenous heparin was administered throughout the procedure, and the activated clotting time was maintained at ≥350 seconds.

An electroanatomic map was created using the CARTO 3 system and an OCTARAY mapping catheter (Biosense Webster Inc.) and then merged with the computed tomography or magnetic resonance imaging scans. The baseline impedance for each VLCC electrode was measured by positioning the catheter in the blood pool. All 10 electrodes of the VLCC were typically activated for ablation, and when electrode overlapping occurred within the PV, the overlapping electrodes were deactivated. Catheter-tissue contact was continuously monitored using the TPI function, and the catheter was repositioned as needed to maximize the number of TPI-positive electrodes before PF energy delivery.

PFA was delivered in 3 consecutive applications. For each PV, 6 applications were delivered at the PV ostium and 6 at the PV antrum for a minimum of 48 applications per patient; additional applications were delivered as needed.^4^^,^^18^ At the operator’s discretion, LA posterior wall isolation was performed with PFA and adjunctive radiofrequency ablation (RFA) using a QDOT MICRO catheter (Biosense Webster Inc.) was applied to the cavotricuspid isthmus, superior vena cava, or inducible atrial tachycardia.

After PFA, an LA voltage map was created during atrial pacing to confirm PV isolation. Additional PFA applications were performed if residual PV potentials was observed. Exit block was confirmed by pacing within each PV.

### Laboratory assessment

Blood samples were collected the day before and the morning after the procedure. The following parameters were measured: lactate dehydrogenase (LDH), total bilirubin (T-Bil), haptoglobin, serum creatinine, and hemoglobin. The time interval between procedure completion and postoperative blood collection was also recorded.

AKI was defined according to the Kidney Disease: Improving Global Outcomes (KDIGO) guideline as either a ≥1.5-fold increase in serum creatinine or an absolute increase of ≥0.3 mg/dL compared with baseline.^19^

Hemolysis was assessed using 3 markers: the postoperative-to-preoperative haptoglobin ratio (Hpt ratio = postoperative haptoglobin/preoperative haptoglobin), postoperative LDH, and T-Bil. We selected these markers because these are widely used indicators of intravascular hemolysis in the PFA literature.^6^^,^^8^^,^^10^^,^^11^^,^^20^ The destruction of erythrocytes leads to LDH elevation and increased bilirubin production, while haptoglobin decreases as it binds free hemoglobin. Because haptoglobin levels vary significantly among individuals owing to phenotypic variations, the Hpt ratio was used to normalize these interindividual differences.

### Assessment of catheter-tissue contact with TPI

For every PFA application set, catheter-tissue contact at each VLCC electrode was assessed using the TPI function. For each patient, we counted the cumulative number of VLCC electrode uses delivering PF energy; deactivated electrodes and incomplete applications were excluded.

**Figure 1:**
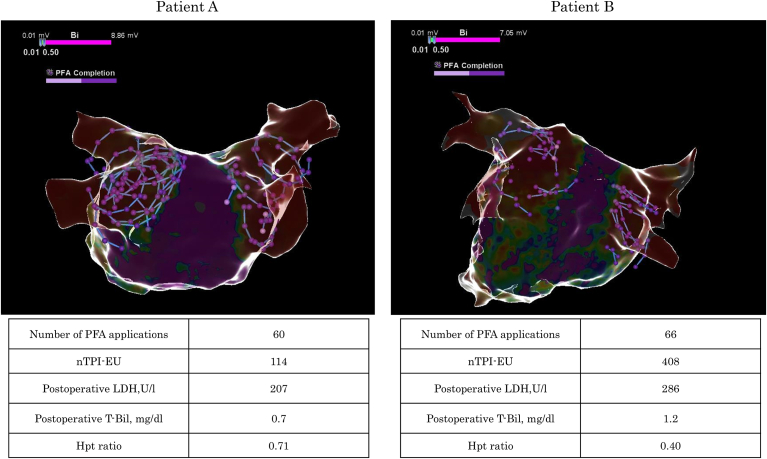
Left atrial voltage maps after pulsed field ablation (PFA) (posteroanterior view) in 2 representative cases. Tags indicate PFA applications delivered from tissue proximity indication–positive electrodes. Hpt ratio = ratio to postoperative-to-preoperative haptoglobin ratio; LDH = lactate dehydrogenase; nTPI-EU = cumulative number of tissue proximity indication–negative electrode uses; T-Bil = total bilirubin.

Using a TPI tag filter, we identified VLCC electrode uses that were TPI-negative at the moment of PFA delivery and summed them to derive the cumulative number of TPI-negative VLCC electrode uses (nTPI-EU) (Figure 1). In addition, for each ablation site, the proportion of TPI-positive electrodes among all activated electrodes was calculated.

### Statistical analysis

Continuous variables are presented as mean ± SD for normally distributed data or as median (interquartile range) for nonnormally distributed data. Between-group comparisons of continuous variables were performed using the Student *t* test or Mann-Whitney *U* test, as appropriate. Categorical variables are expressed as count and percentage and were compared using the χ^2^ test.

Associations between each hemolysis marker and variables such as LA diameter, total number of PFA applications, and nTPI-EU were assessed using the Pearson correlation coefficient.

Statistical significance was defined as a 2-sided *P* value of <.05. All analyses were performed using SPSS Statistics software version 25.0 (IBM Corporation).

## Results

### Patient characteristics

Overall, 20 patients were included in the study. The mean age was 67.4 ± 9.9 years, and 11 patients (55.0%) were men. Paroxysmal AF was present in 9 patients (45.0%), persistent AF in 9 patients (45.0%), and long-standing persistent AF in 2 patients (10.0%). Four patients (20.0%) had a history of structural heart disease: 1 (5.0%) had dilated cardiomyopathy, 1 (5.0%) had hypertrophic cardiomyopathy, and 2 (10.0%) had severe mitral regurgitation. Five patients (25.0%) had a history of chronic heart failure. On echocardiography, the mean LA diameter was 40.4 ± 8.2 mm and the mean left ventricular ejection fraction was 57.9% ± 8.5% (Table 1).

**Table 1:** Patients’ characteristics (N = 20)

Characteristic	Value
Age (y)	67.4 ± 9.9
Men	11 (55.0)
Body mass index (kg/m^2^)	24.1 ± 3.9
Comorbidities	
Hypertension	8 (40.0)
Diabetes mellitus	2 (10.0)
Heart failure	5 (25.0)
Stroke	2 (10.0)
Underlying heart disease	4 (20.0)
History of arrhythmia	
Paroxysmal atrial fibrillation	9 (45.0)
Persistent atrial fibrillation	9 (45.0)
Long-standing persistent atrial fibrillation	2 (10.0)
Echocardiographic measurements	
Left atrial diameter (mm)	40.4 ± 8.2
Left ventricular ejection fraction (%)	57.9 ± 8.5

Values are presented as mean ± SD or n (%).

### Procedural data

Table 2 summarizes the procedural data. LA posterior wall isolation with PFA was performed in 2 patients (10.0%). Cavotricuspid isthmus line ablation was performed in 15 patients (75.0%) and superior vena cava isolation in 1 patient (5.0%). In 1 patient (5.0%), perimitral atrial flutter was induced and a mitral isthmus lateral line was created using RFA.

**Table 2:** Procedural data (N = 20)

Ablation characteristic	Value
Contrast medium (mL)	24 (0–36)
Intraprocedural fluid volume (mL)	738 (622–919)
Total procedure time (min)	96 (80–110)
Total fluoroscopy time (min)	20.3 (17.6–27.4)
Additional ablation	
LA posterior wall isolation (PFA)	2 (10.0)
Cavotricuspid isthmus line (RFA)	15 (75.0)
Superior vena cava isolation (RFA)	1 (5.0)
Lateral mitral isthmus line (RFA)	1 (5.0)
PFA application data	
Number of PFA applications per patient	78.3 ± 20.0
Cumulative number of VLCC electrode uses delivering PF energy	772.9 ± 204.1
Cumulative number of TPI-negative VLCC electrode uses delivering PF energy (nTPI-EU)	438.8 ± 238.1
Proportion of TPI-positive VLCC electrodes	
Total (%)	45.1 ± 16.7
Left superior PV (%)	62.1 ± 28.8
Left inferior PV (%)	51.7 ± 24.0
Left PV carina (%)	25.1 ± 22.9
Left common PV (%)	60.0 ± 12.2
Right superior PV (%)	58.1 ± 25.7
Right inferior PV (%)	50.5 ± 21.2
Right PV carina (%)	29.4 ± 20.3
LA posterior wall (%)	16.9 ± 17.7

Values are presented as mean ± SD, median (interquartile range), or n (%).

LA = left atrial; nTPI-EU = cumulative number of tissue proximity indication–negative electrode uses; PF = pulsed field; PFA = pulsed field ablation; PV = pulmonary vein; RFA = radiofrequency ablation; TPI = tissue proximity indication; VLCC = variable-loop circular catheter.

The mean number of PFA applications per patient was 78.3 ± 20.0. The cumulative number of VLCC electrode uses in which PF energy was delivered was 772.9 ± 204.1, of which 438.8 ± 238.1 were classified as nTPI-EU. The mean proportion of TPI-positive VLCC electrodes per patient was 45.1% ± 16.7%. This proportion was lower in the PV carina and the LA posterior wall than in the PV ostium.

### Laboratory data

The postoperative blood sample was collected a median of 19.3 hours (interquartile range 16.4–20.2 hours) after the procedure. The pre- and postoperative laboratory results are summarized in Table 3. Both LDH and T-Bil were significantly elevated postoperatively compared with preoperative values, whereas haptoglobin were significantly decreased (*P* < .01 for all). The mean Hpt ratio was 0.46 ± 0.19.

**Table 3:** Laboratory data

Variable	Before the procedure	After the procedure	*P*
LDH (U/L)	199.6 ± 28.6	253.2 ± 48.2	<.01
T-Bil (mg/dL)	0.8 ± 0.3	1.4 ± 0.6	<.01
Haptoglobin (mg/dL)	108.8 ± 60.0	51.0 ± 39.4	<.01
Serum creatinine (mg/dL)	0.9 ± 0.2	0.9 ± 0.2	.18
Hemoglobin (g/dL)	14.1 ± 1.6	12.7 ± 1.5	<.01

Values are presented as mean ± SD.

LDH = lactate dehydrogenase; T-Bil = total bilirubin.

Serum creatinine showed no significant difference between pre- and postoperative measurements (*P* = .18). One patient developed AKI on postoperative day 1 (serum creatinine increased from 0.68 to 1.00 mg/dL); this patient had undergone 129 PFA applications and 1188 nTPI-EU. Dialysis was not required, and serum creatinine returned to baseline within several days.

Although postoperative hemoglobin decreased significantly compared with its preoperative value (*P* < .01), no patient experienced a decrease of >3 g/dl or blood transfusion.

**Figure 2:**
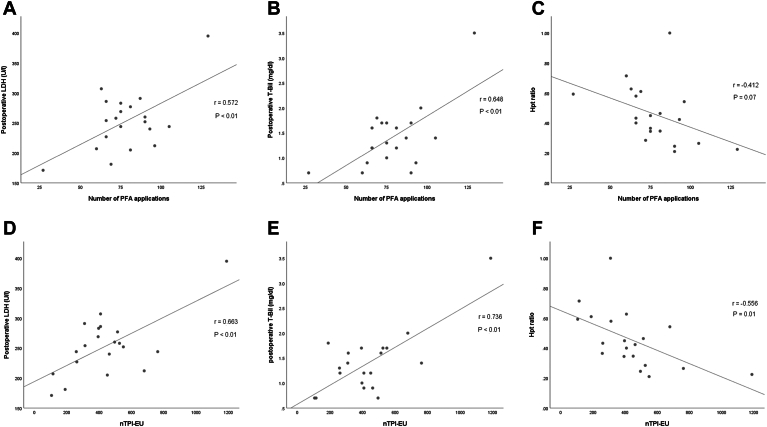
Correlations of postoperative lactate dehydrogenase (LDH), total bilirubin (T-Bil), and postoperative-to-preoperative haptoglobin ratio (Hpt ratio) with (**A–C**) the number of pulsed field ablation (PFA) applications and (**D–F**) the cumulative number of tissue proximity indication–negative electrode uses (nTPI-EU).

The correlation coefficients between clinical variables and hemolysis markers are presented in Table 4. Postoperative LDH was significantly positively correlated with the number of PFA applications (*r* = 0.572; *P* < .01) and nTPI-EU (*r* = 0.663; *P* < .01) (Figure 2A and 2D). It was also significantly higher in patients who underwent LA posterior wall isolation (*P* = .03).

**Table 4:** Univariable analysis of the correlation between variables and hemolysis markers

Variable	LDH		T-Bil		Hpt ratio	
	Correlation coefficient (*r*)	*P*	Correlation coefficient (*r*)	*P*	Correlation coefficient (*r*)	*P*
LA diameter	0.219	.35	0.623	<.01	−0.471	.04
Number of PFA applications	0.572	<.01	0.648	<.01	−0.412	.07
nTPI-EU	0.663	<.01	0.736	<.01	−0.556	.01

Hpt ratio = ratio to postoperative-to-preoperative haptoglobin ratio; LA = left atrial; LDH = lactate dehydrogenase; nTPI-EU = cumulative number of tissue proximity indication–negative electrode uses; PFA = pulsed field ablation; T-Bil = total bilirubin.

Postoperative T-Bil was positively correlated with LA diameter (*r* = 0.623; *P* < .01), number of PFA applications (*r* = 0.648; *P* < .01), and nTPI-EU (*r* = 0.736; *P* < .01) (Figure 2B and 2E).

The Hpt ratio did not correlate significantly with the number of PFA applications (*r* = −0.412; *P* = .07) but correlated negatively with LA diameter (*r* = −0.471; *P* = .04) and nTPI-EU (*r* = −0.556; *P* = .01) (Figure 2C and 2F).

## Discussion

nTPI-EU showed significant correlations with all hemolysis markers (LDH, T-Bil, and Hpt ratio). We demonstrate in humans that hemolysis is associated with PFA delivery from noncontact catheter electrodes, as assessed using the TPI function.

In a swine model, hemolysis was significantly greater when the catheter was floating in the blood pool than when it was in direct contact with the tissue.^13^, ^14^, ^15^ This effect is thought to result from the uneven distribution and excessive localization of electric field intensities, which can cause erythrocyte rupture.^21^ However, previous studies have primarily examined binary conditions, either full contact or complete noncontact between the catheter and tissue. In contrast, the present study suggests that hemolysis may still occur when only a portion of the catheter electrodes is not in adequate contact with myocardial tissue during PFA delivery. Figure 1 illustrates 2 representative cases. Patients A and B underwent similar numbers of PFA applications (60 and 66, respectively); however, their nTPI-EU values were markedly different (114 vs 408). Patient B, who had the higher nTPI-EU, exhibited more pronounced postoperative hemolysis (LDH 286 U/L; T-Bil 1.2 mg/dL; Hpt ratio 0.40) than did patient A (LDH 207 U/L; T-Bil 0.7 mg/dL; Hpt ratio 0.71). These findings suggest that minimizing hemolysis requires not only limiting the total number of PFA applications but also ensuring that as many catheter electrodes as possible are in adequate contact with the myocardium during PFA delivery.

Hemolysis is more pronounced when PFA is performed beyond the PVs (eg, roofline, LA posterior wall, mitral isthmus, or right atrium) compared with PV isolation alone.^3^^,^^6^^,^^8^ In our cohort, PFA delivered outside the PV ostia, particularly at the LA posterior wall, was associated with a lower proportion of TPI-positive VLCC electrodes. This may be due to the inability of the VLCC to fully conform to complex anatomical structures beyond the PVs. As a result, both an increased number of PFA applications and greater involvement of noncontact catheter electrodes may increase the risk of hemolysis.

Regarding the observed postoperative decline in hemoglobin, it remains unclear whether this was directly caused by PFA. Similar reductions in hemoglobin levels have been reported even in patients undergoing RFA.^6^^,^^10^ Therefore, the contribution of PFA-induced hemolysis to postoperative hemoglobin decline is likely minimal, with factors such as dilution from intraprocedural fluid administration considered more likely to be responsible.

In this study, only 1 case of AKI occurred, precluding a detailed analysis of its risk factors. However, in that patient, not only was the total number of PFA applications high but the nTPI-EU was also markedly elevated. This raises the possibility that hemolysis resulting from energy delivery through noncontact electrodes may have contributed to the development of AKI.

### Limitations

This study had some limitations. First, this was a retrospective single-center study with a small sample size (N = 20). Second, hemolysis markers were assessed only once postoperatively, in the morning after the procedure, and variations in the timing of blood sampling may have affected the results. Third, procedures were performed by 3 operators; however, because of the limited sample size, we did not perform operator-level analyses and residual interoperator variability cannot be excluded. Fourth, although TPI status was used as a surrogate for catheter-tissue contact, it does not necessarily guarantee consistent contact. TPI signals can fluctuate because of respiratory and cardiac motion, reflecting intermittent loss of contact. Because procedures were performed under deep sedation rather than general anesthesia, spontaneous respiration or coughing may have further contributed to such flickering. Intracardiac echocardiography has recently used to directly assess catheter-myocardial contact; therefore, we believe that further studies using intracardiac echocardiography are warranted.^22^

## Conclusion

This study demonstrated that a higher cumulative number of VLCC electrodes lacking adequate catheter-tissue contact at the time of PFA delivery was significantly associated with hemolysis markers. These findings suggest that, in addition to limiting the total number of PFA applications, optimizing catheter-tissue contact, particularly by minimizing the number of noncontact catheter electrodes, is essential for reducing the risk of hemolysis.

## Disclosures

The authors declare that they have no conflicts of interest.
